# State of the Art of Non-Invasive Electrode Materials for Brain–Computer Interface

**DOI:** 10.3390/mi12121521

**Published:** 2021-12-08

**Authors:** Haowen Yuan, Yao Li, Junjun Yang, Hongjie Li, Qinya Yang, Cuiping Guo, Shenmin Zhu, Xiaokang Shu

**Affiliations:** 1 State Key Laboratory of Metal Matrix Composites, Shanghai Jiao Tong University, Shanghai 200240, China; yuanhw0619@sjtu.edu.cn (H.Y.); woniujun@sjtu.edu.cn (J.Y.); lihongjie0215@sjtu.edu.cn (H.L.); yangqinya@sjtu.edu.cn (Q.Y.); guocuiping@sjtu.edu.cn (C.G.); smzhu@sjtu.edu.cn (S.Z.); 2 School of Mechanical Engineering, Shanghai Jiao Tong University, Shanghai 200240, China

**Keywords:** brain-electrode interface, EEG electrodes, dry electrodes, wet electrodes, semi-dry electrodes

## Abstract

The brain–computer interface (BCI) has emerged in recent years and has attracted great attention. As an indispensable part of the BCI signal acquisition system, brain electrodes have a great influence on the quality of the signal, which determines the final effect. Due to the special usage scenario of brain electrodes, some specific properties are required for them. In this study, we review the development of three major types of EEG electrodes from the perspective of material selection and structural design, including dry electrodes, wet electrodes, and semi-dry electrodes. Additionally, we provide a reference for the current chaotic performance evaluation of EEG electrodes in some aspects such as electrochemical performance, stability, and so on. Moreover, the challenges and future expectations for EEG electrodes are analyzed.

## 1. Introduction

In 2014, a young paralyzed boy completed a kick-off ceremony with the help of bionics technology [1]. It shocked many people that a paralyzed child could complete such actions. The whole system was realized because of the brain–computer interface (BCI).

BCI is a new technology with multidisciplinary connections including materials, neuroscience, signal processing, and so on. The nervous activity produced by emotion swings was applied to drive the external application work. The concept of BCI was proposed by Jacques Vidal in 1973 [2]. Additionally, in 1977, Vidal achieved the utilization of the brain to directly control the movement of a two-dimensional point in a maze on the computer screen by ERP (event-related potentials) [3]. The first BCI international meeting in 1998 led to the proposal of a detailed definition: brain–computer interfaces give their users communication and control channels that do not depend on the brain’s normal output channels of peripheral nerves and muscles [4].

**Figure 1:**
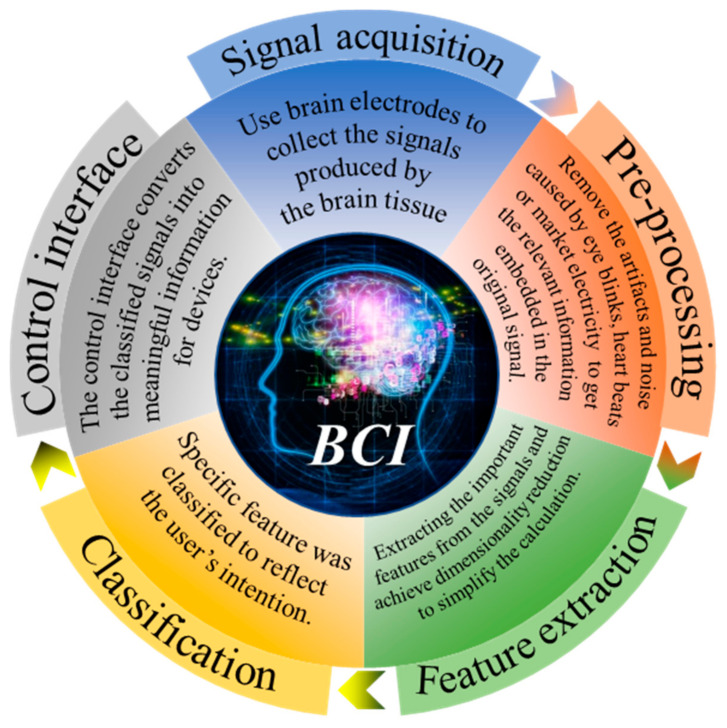
Five components of BCI [21,22,23,24,25,26].

BCI was originally designed to facilitate the disabled. Nowadays, BCI can be used in many fields, including medicine, entertainment, industry, and other applications. The medical field is currently the largest area where BCI is applied [5,6,7]. For example, it can be used as alternative communication and environment control to enable amyotrophic lateral sclerosis (ALS) patients to communicate [8] or in post-stroke motor rehabilitation [9,10]. Mental illnesses such as depression can also be treated with BCI [11]. In addition, BCI can also test the difficulty of working in different environments, such as predicting the difficulty of playing Tetris by EEG activity [12], evaluating the pressure load changes of surgeons during simple and complex surgical training tasks [13], detecting drivers’ fatigue degree during driving [14], and evaluating the sustained attention of real word classroom students [15]. BCI also has some preliminary applications in the field of games [16], such as using EEG to control archery [17] and the realization of multiplayer online VR racing games through BCI [18]. In addition, there are also games combined with medical treatment-based training systems used to analyze or improve children’s attention deficit and hyperactivity disorder in the process of reading [19]. As the first BCI application, BCI spellers have been widely used in recent years, as shown in this review [20]. However, most of these applications can only be achieved in a laboratory. From these technologies, we can see countless possibilities and enormous challenges. As shown in Figure 1, the acquisition of the signal is the first step and the most important part of BCI.

The quality of the signals directly determines the difficulty of the subsequent work and the final effect of the whole system. The brain electrodes remain an important technological challenge and the current focus of BCI research. At present, BCI signal sensor modalities mainly include implanted microelectrodes, ECoG (electrocorticography), intravascular electrodes, EEG (electroencephalogram), fMRI (functional magnetic resonance imaging), fNIRS (functional near-infrared spectroscopy), fTCD (functional transcranial doppler), MEG (magnetic encephalography), and PET (positron emission tomography) [27]. The electrical sensor modalities are commonly used at present.

Invasive electrodes were first applied to neurology examinations resulting from their most perfect signal because they can penetrate specific regions of the brain parenchyma to measure neuronal electrophysiological activity. The shorter distance between the neurons and the electrodes suggests better brain electrical signals due to less interference in the signal transmission path. Therefore, many BCIs in the early stage used invasive electrodes as electrical signal acquisition methods. The materials used for invasive electrodes have developed from metal wire [28,29,30,31,32,33] to silicon-based microprobes [34,35,36,37,38]. At present, conductive materials located on a substrate made of soft materials with good biocompatibility especially biomaterials such as alginate, chitosan, collagen, and cellulose have become the ideal choice [39,40]. The final failure of invasive recording is usually attributed to the coupling of both abiotic and biotic perspectives [33]. The abiotic factor implies the electrode will eventually expire. Large variation in electrode impedance and appearance, as well as structural changes on the electrodes’ recording surface, were observed after several weeks of implanting. Biotic problems occur even at the very beginning of the implantation, including, but not limited to, direct lesions or induced inflammation.

At present, although the basic theory, preparation technology, and performance modification of invasive electrodes have been studied for a long time and several strategies have been considered to improve microelectrode performance including those that improve the stability of conducting and insulating materials, the fabrication process and the electrode integration with the surrounding tissues and their stability and durability are still issues worth considering. Premature electrode failure and possible neuro-inflammation hinder their long-term application, especially for the brain–machine interface.

Semi-invasive electrodes for measuring ECoG are also called injectable electrodes, which are less invasive than intra-parenchymal microelectrodes. Injecting the net-shape electrodes into a specific position of brain tissue will decrease pain and improve the quality of the recording signal. The mesh structure makes the electrode softer than traditional structures such as paper, needle array, and so on. As a result, the electrodes can adhere more closely to the brain tissue [41,42,43,44]. Semi-invasive electrodes overcome some disadvantages of invasive electrodes; they are less invasive and cause less inflammation. As a result, they are more suitable as a means of long-term ECoG recording. However, their special structure brings out some specific problems. The rheological characteristics of semi-invasive electrodes have been studied, but still not in-depth. Some problems such as the fluidity of the mesh in the needle and the entanglement of the whole structure after injection still hinder the practical application. Moreover, even minimally invasive surgery also scares users.

Factors such as high surgery costs, complicated preparation, and, especially, the great harm to brain health make the use of these electrodes feasible only in animals or in a laboratory. For those who do not need to regard BCI as a lifesaver, both invasive methods and semi-invasive methods are not acceptable. Commercial invasive electrodes for BCI have been hardly available thus far. The EEG electrodes without invasiveness have become the type with the best commercial prospects.

In this review, we give an overview of the development of traditional EEG brain electrode materials and propose an exhaustive, detailed summary of the materials currently used in brain electrodes. In the light of different structures and working methods, we classify the EEG brain electrodes into the following types: wet electrodes, dry electrodes, capacitive electrodes, and semi-dry electrodes. In addition, we review and summarize some previous evaluation criteria of EEG electrode performance.

## 2. EEG Electrodes

**Figure 2:**
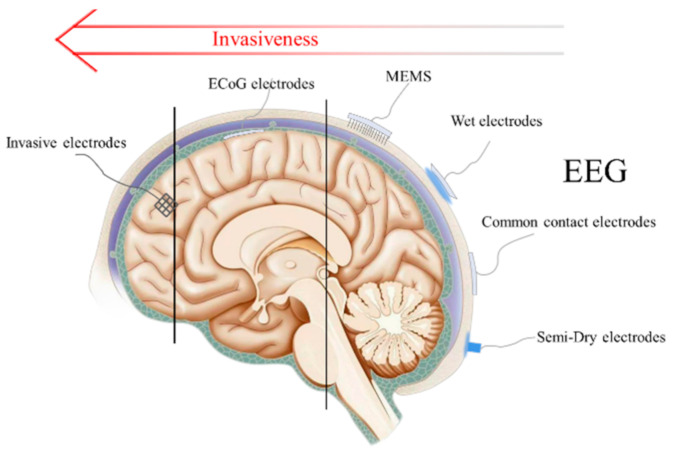
Schematic diagram and invasiveness degree of EEG electrodes, ECoG electrodes and invasive electrodes.

The non-invasive technique is the commercial signal acquisition method currently used in BCI. By placing brain electrodes on the surface of the scalp, an electroencephalogram (EEG) can be recorded without invading brain tissue. What follows is that the EEG signal amplitude will be much smaller than that which was recorded by invasive electrodes due to the interference caused by the cranium, skin tissue, and hair. Because of its low trauma, or even non-trauma to the human body, non-invasive electrodes have gradually become the mainstream application of BCI electrodes. As shown in Figure 2, there are three main types of non-invasive electrodes including wet electrodes, dry electrodes, and emergent semi-dry electrodes according to different conditions of use.

### 2.1. Principle of EEG Acquisition

**Figure 3:**
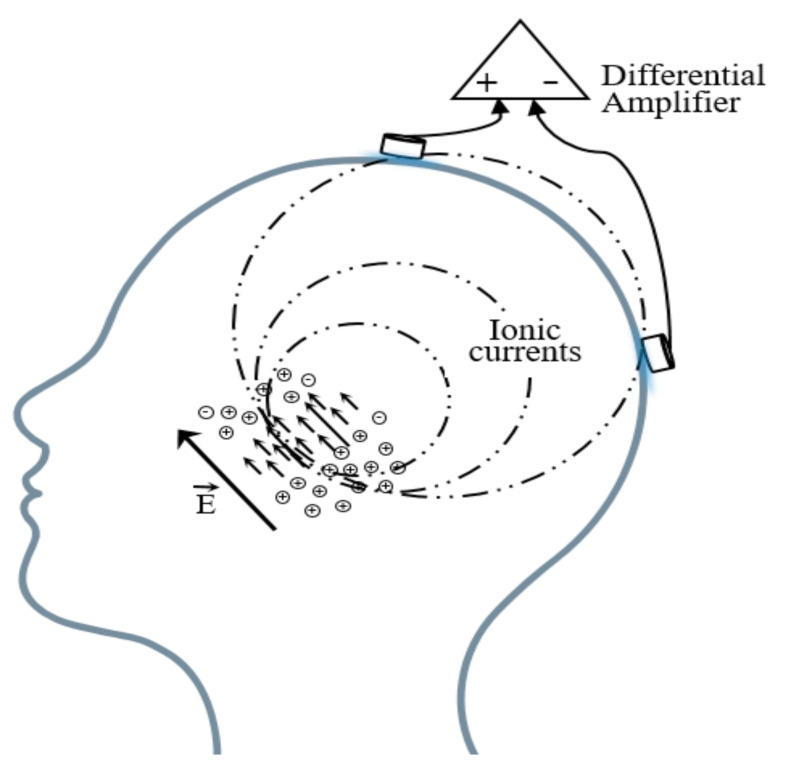
Schematic diagram of the EEG acquisition.

Brain electric activity mainly comes from postsynaptic potentials. The brain electrical current is generated by the flow of Na^+^ K^+^ Ca^++^ Cl^−^, leading to the electric dipole. The electrical activity signal of a single neuron is so weak that only the sum of the electrical activities of numerous neurons can be detected [45]. EEG is the potential difference between two electrodes put on the scalp as shown in Figure 3.

**Figure 4:**
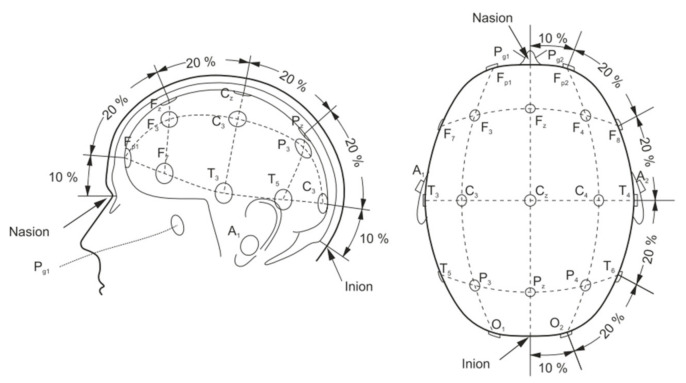
Electrode placement over scalp according to the international 10–20 system [46].

A basic EEG acquisition system usually consists of three electrodes including an active electrode (A), a reference electrode (R), and a grounding electrode (G). The AG potentials and RG potentials are then connected to a differential amplifier to eliminate environmental electrical activity. Therefore, EEG not only reflects the electrical activity on where the active electrode is placed but also the potential difference between the location of the active electrode and the reference electrode. According to the recommendations of the American Electroencephalography Society, active electrodes are not randomly placed on the brain but are based on the international 10–20 system (as shown in Figure 4) [46].

The amplitude of the scalp EEG is usually less than 100 μV. In daily applications, although the differential amplifier circuit can remove most of the noise, the residual noise will still distort the result due to the difference between the electrodes and the limitation of the amplifier performance. Generally, the desired signal can be separated from the background noise by averaging signals. However, for applications such as BCI that require an immediate response, increasing the number of measurement times is not a reliable choice. After the signal is collected, there will be pre-processing for the signal such as filtering, but filtering will distort the result in many aspects. For instance, the filter may change the start and end time of a waveform and may cause the original single-phase transform to become multiphase. Therefore, it is very important to obtain clear data at the electrode directly.

In general, contact impedance will directly affect EEG collection. The well-known resistance is only suitable for the concept of direct current. Additionally, in the acquisition of an EEG, the potential constantly changes with time, so the inductance and capacitance will also hinder the current, which is collectively defined as impedance. In the context of EEG recording, impedance is typically measured by passing a small 10 Hz current closed to the EEG frequency between two electrodes [47].

**Figure 5:**
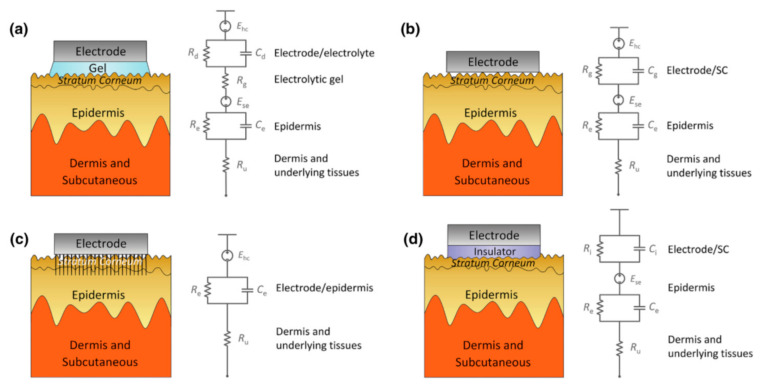
Schematics and corresponding electrode–skin interface models for EEG electrodes. (**a**) Wet electrodes, (**b**) MEMS electrodes, (**c**) non-contacted electrodes, and (**d**) common-contact dry electrodes [50].

Impedance has the following effects on signal acquisition. Firstly, the high impedance will significantly reduce the common-mode rejection ratio (CMRR), which determines the ability to suppress the common noise of the working electrode and the reference electrode caused by lights, video displays, and so on. However, the CMRR can be enhanced by increasing the input impedance of the amplifier because the larger the input impedance, the closer the input voltage obtained by the amplifier circuit to the signal source voltage. However, the input impedance ought not to reach an exceedingly high level because the current will become susceptible to any electromagnetic disturbances. Therefore, the input impedance also has a certain adjustment range, and the skin contact impedance should be reduced as much as possible. Secondly, the high impedance will cause a great drift in the skin potential. This noise cannot be eliminated by common-mode suppression such as human body movement and environmental electrical noise. The skin potential of each electrode’s contact part, in turn, depends on factors such as the thickness of the skin, the number of sweat glands and hair follicles, and the degree of skin hydration [48,49]. Any difference in the conductance of the skin will lead to a different voltage offset for each electrode. Low-contact impedance will condense the skin potential drift and abate the noise. Figure 5 clearly describes the impedance composition of different electrode–skin interface models under different EEG electrodes.

### 2.2. Wet Electrodes

As the name suggests, wet electrodes need a conductive paste dissolved in the stratum to decrease impedance so that there is better contact between the electrodes and the skin. Due to the decrease in impedance, the quality of the signal collected by wet electrodes is much better than that of dry electrodes. In addition, wet electrodes are not sensitive to motion artifacts. As a result, wet electrodes were called the “gold standard”, and one way to assess the quality of the signal collected by “homemade electrodes” is to compare it with the signal collected by wet electrodes to obtain the correlation [51,52,53].

Wet electrodes have developed for a long time. The most popular materials for wet electrodes are Ag/AgCl [54,55,56,57,58] because they have wide availability, are sufficiently low impedance, and have a more stable contact potential consequently giving rise to better noise behavior than other metals when contacting with the electrolyte. In addition, some researchers have also taken the gold cup electrode as the comparison object [59,60,61].

In fact, conductive gel plays a more important role in wet electrodes. It always consists of water, an ionic salt, surfactant, thickener, and bactericide/fungicides [62]. Some researchers have also modified the conductive gel to surmount the defect of inconvenient use. Pedrosa et al. [63] proposed a novel alginate-based hydrogel as an alternative to traditional EEG electrolytic gels. Unlike traditional gels, the alginate polymer subsequently forms a solid hydrogel after being injected into the electrode cavity that exists in most EEG cap systems, avoiding gel leaking and cleaning procedures. The EEG tests showed no considerable differences between signals acquired with the hydrogel and the commercial electrolytic gel during the same signal acquisition in terms of amplitude, signal shape, power spectral density, and signal-to-noise ratio. Researchers who have used the wet electrode as their own EEG acquisition method have usually focused on the following signal processing and signal feature extraction rather than the electrode itself. Therefore, few articles focus on the improvement of the conductive adhesive. Both in the medical and BCI fields, wet electrodes are currently the most balanced choice regarding signal quality and safety. Even though many efforts have been made to improve the performance of dry electrodes, the quality of the signal recorded by wet electrodes is much better compared to that collected by a dry electrode.

However, wet electrodes also have many disadvantages: a long-term preparation is required before use, and the pre-treatment steps include grinding the stratum corneum and hair removal, which will greatly increase the user’s discomfort. Additionally, some ingredients, such as propylparaben, were pointed out as leading to the presence of parabens in breast cancer tumors, which is an endocrine disruptor due to its estrogenic effect [64]. Furthermore, if the distance between the electrodes is too close, the conductive paste will come into contact and lead to a short circuit [65,66]. Owing to water evaporation as well as the self-curing ability of skin tissue, the conductivity of the conductive paste will continue to decline over time. Nonetheless, wet electrodes are widely used in clinical and scientific research because of their good signal quality and low invasiveness. The shortcomings of wet electrodes and the need for professional assistance severely limit their commercial value in daily life.

### 2.3. Dry Electrodes

To overcome problems in their daily use, a variety of dry electrodes have been proposed. Dry electrodes are those that do not use any liquid conductive medium between the skin and electrode surface. However, in fact, the electrode is not completely dry; there will be some sweat as electrolytes during use. Additionally, depending on the contact methods with the scalp, there are three kinds of dry electrodes: MEMS electrodes, non-contact electrodes, and ordinary-contact electrodes.

#### 2.3.1. MEMS Dry Electrodes

MEMS electrodes are different from the invasive electrodes mentioned in Part 1. The microneedles of MEMS dry electrodes only puncture the stratum corneum, which is composed of dead cells and does not harm brain tissue. From the equivalent circuits, it is very clear that the MEMS dry electrodes avoid the impedance caused by the stratum corneum. Furthermore, since the pin directly pierces the scalp, electrodes will be better fixed, basically avoiding the generation of motion artifacts.

The bioelectric signal quality obtained by MEMS is better compared to other dry electrodes, and the electrochemical noise is lower [67]. In addition, this method eliminates the need for skin treatment and avoids the tedious preparation steps. There are main three types of microneedle array dry electrodes, including silicon substrate microneedle array dry electrodes, metal microneedle array dry electrodes, and polymer microneedle array electrodes.

The silicon processing technique was initially proposed a long time ago, and now there are three main preparation methods by which to fabricate EEG MEMS electrode substrates [68,69,70,71,72,73], including self-stop etching of doped silicon, deep reactive ion etching, and isotropic and anisotropic reactive ion etching. After the etching, conductive materials are needed for the signal recording. Some metal particles, such as Ti/Pt [70], Ti/Ag [72], and Ag/AgCl [73], are distributed on the substrate by electrodeposition or other surface treatments. Although the manufacturing process of silicon is very mature, it has poor biocompatibility, and microneedles are brittle, which makes them easily break when they penetrate the skin. The application of silicon is thus limited.

In addition to silicon-based microneedle array dry electrodes, there are metal microneedle array dry electrodes and polymer microneedle array dry electrodes, both of which have their own advantages and disadvantages. Metal materials have good mechanical properties and electrical conductivity, so they can penetrate the cuticle better than polymer materials. Metal microneedle array electrodes materials mainly include Ti/Au [74], Cu [75,76], stainless steel [77], and IrO_2_ [78] as their composition can effectively record bioelectric signals. These materials all have good biocompatibility and are easily obtained. However, there are still some problems with metal microneedle array electrodes. The rigid substrate makes it difficult to come into close contact with the skin, so the comfort level will be poor, and the bad contact will cause unstable signals. The processing technology is still not perfect, which leads to low processing accuracy. Polymer microneedle array dry electrodes have the characteristics of good biocompatibility and low cost. Polymers that are used to prepare microneedle array dry electrodes include PDMS, SU-8, parylene C, epoxy resin, polymethyl methacrylate, and polyimide [60,79,80,81,82,83,84,85]. The surface of the polymer microneedles prepared from the above materials also needs to ensure electrode conduction through surface deposition and electroplating metal materials including Au Ag Pt, and Ni, etc. However, polymer microneedle array dry electrodes also have some disadvantages. The polymer has a low hardness, and it cannot easily penetrate the stratum corneum.

**Figure 6:**
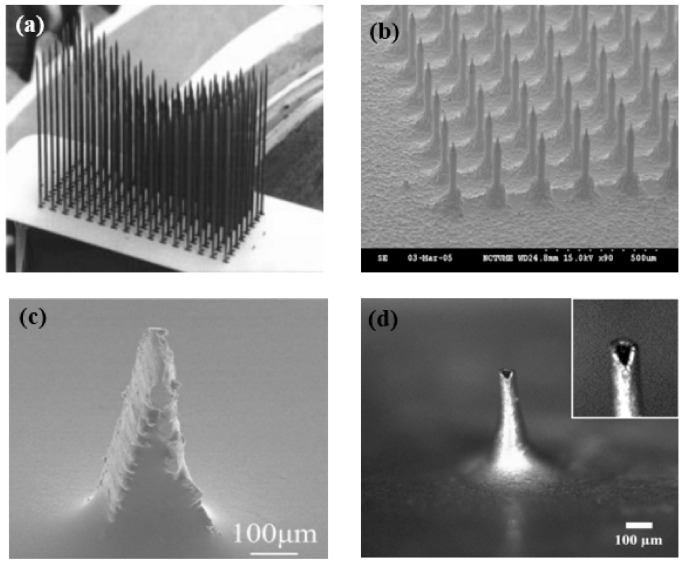
Typical MEMS electrodes and capacitive electrodes. (**a**,**b**) Silicon-based microneedle array dry electrodes [68,70]. (**c**) Metal microneedle array electrodes [74]. (**d**) Polymer-based microneedle array dry electrode [81].

At present, a commonly used method is to combine the flexible substrates and the hard microneedle array. This method can not only ensure that the dry electrode can be close to the scalp but also allows the microneedle to penetrate the stratum corneum smoothly. The manufacturing process of microneedle array dry electrodes is becoming increasingly sophisticated, and its application in bioelectrical signal acquisition is gradually increasing, but this electrode does not have the features of being lightweight or integrated at present. In addition, the microneedle piercing the skin will still make users uncomfortable and can cause a risk of infection. As shown in Figure 6, no matter what kind of MEMS electrode, it will have a very sharp structure. With the continuous application of new materials and the constant improvement of the structure, microneedle array dry electrodes will be a good choice for BCI to collect electrical signals.

#### 2.3.2. Non-Contacted Electrodes

Non-contacted electrodes are similar to capacitive coupling directly to the skin, which can collect bioelectric signals without touching the skin. Therefore, they are also called capacitive electrodes. Because of the non-contact performance, capacitive electrodes can overcome the defect of other electrodes that are only able to be used on hairless sites.

Non-contact electrodes contain a metal plate electrode and an active circuit. The active circuit must provide an ultra-high input impedance to avoid signal attenuation because the metal electrode can be viewed as an ultra-small capacitor. The input impedance of the amplifier should reach a high level that is four orders of magnitude greater than that of traditional wet electrodes. As a result, this places high demands on the amplifier. Lee et al. [86] designed a capacitive electrode composed of upper and lower PDMS layers, a shield plate, an electrode plate, and an adhesive PDMS layer. The electrode plate was fabricated in five layers: 30 μm polyimide, 30 nm titanium, 10 μm Cu, 30 μm Ni, and 100 nm Au. Additionally, the electrode plate was sandwiched by the PDMS layers. In the eye-closed and eye-open tests, the alpha rhythm had correlations of 0.91 and 0.83 with conventional electrodes. Liu et al. [87] designed a non-contact signal acquisition system with a diameter of 25 mm and made of a flexible printed circuit. The electrode had a two-layer structure. The bottom layer was composed of a copper-filled capacitive sensing plate and a shielding ring to avoid external interferences. The top layer was made of Cu and connected with the outer ring to improve the shielding outcomes. The electrical components were placed on the flexible printed circuit board instead of the back of the non-contact electrode to ensure optimal coupling and comfort. In general, non-contact electrodes cannot be fixed completely, which results in serious motion artifacts. In this regard, Chen et al. [88] proposed a non-contact electrode containing an active circuit and a metal plate made of Cu. The thickness and diameter were 2 mm and 20 mm, respectively. In addition, the dry electrode had an adaptive mechanical design so that the influence of motion artifacts could be reduced greatly.

In addition, some researchers have made some improvements regarding lightweight designs. Alireza et al. [89] proposed a wearable wireless device for EEG monitoring and analysis. The substrate was made of a four-layer polyimide on which the recording, quantization, and motion artifact removal circuitries were implemented. The whole wearable solution with the battery weighed 9.2 g. Due to its lightweight design, the electrode could not only be used in a laboratory but also for ambulatory EEG recording. The eight channels were sufficient to ensure that brain neural activity could be recorded with a reasonable spatial resolution.

From the above references, there is no doubt that copper is a commonly used capacitor material because of its accessibility, good molding property, and good conductivity. The electrode does not require a strict performance for materials. Compared to wet electrodes, the correlation between the two electrodes is about 90% and can meet the basic requirements for application. In addition, from Alireza’s work [89], non-contracted electrodes had the characteristic of being lightweight (9.2 g). The biggest obstacle to the development of capacitive electrodes is the motion artifact caused by the high impedance and phase shift [90].

#### 2.3.3. Common-Contact Dry Electrodes

Common-contact dry electrodes are the most convenient type since they can directly contact the scalp, but do not penetrate the cuticle or require skin pre-treatment. The existence of hair will hinder the good contact between the electrode and the scalp, which usually requires some special structures for the electrodes to make themselves pass through the hair, such as being finger shaped. For the sake of comfort and high geometric conformity between the sensor and the irregular scalp surface, there is always a spring structure at the end of the probe to cushion the pressure on the scalp. Metal with good conductivity and biocompatibility is usually used for this special structure.

**Figure 7:**
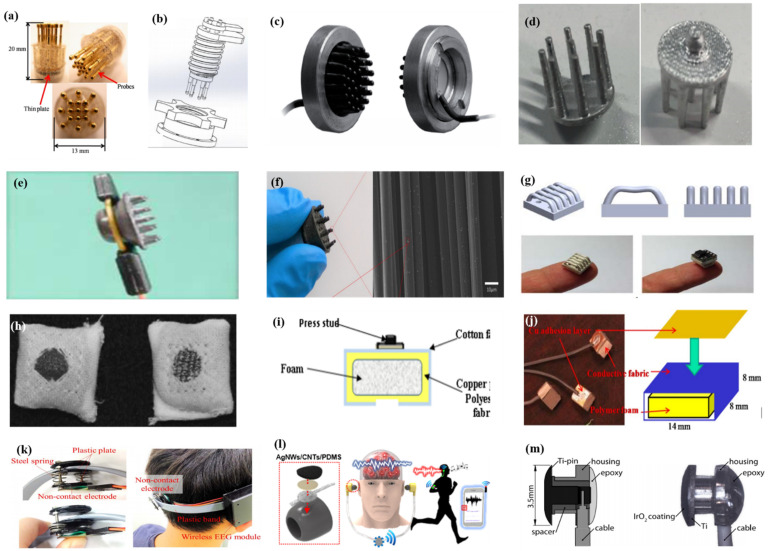
Typical common-contact electrodes. (**a**–**d**) Finger-shaped electrodes [66,91,92,93]; (**e**,**f**) brush-like electrodes [94,96]; (**g**) 3D-printed electrodes [97]; (**h**–**j**) textrodes [100,101,102]; (**k**) non-contacted electrodes [88]; (**l**,**m**) multi-function electrodes [107,108].

As described by Liao et al. [66] in this article, BeCu was used as the electrode column, and Au with good biocompatibility was attached to the contact area between the electrode column and the scalp. A total of 17 probes were next inserted into the thin Cu plate that served as the flexible substrate of the sensor (Figure 7a). Therefore, a good EEG signal can be obtained without skin preparation. Similarly, Liu et al. [91] proposed a spring-type electrode, in which a platinum nanoporous layer was attached to six probes of the electrode so as to decrease contact impedance and enhance conductivity (Figure 7b). Finally, the contact impedance of the dry EEG sensors, which were attached to the different sites, was below 20 kΩ. In the resting-state EEG measurement, the correlations compared to wet electrodes ranged from 0.8179 to 0.9677. Furthermore, coating conductive materials on polymer surfaces can take advantage of the softness of polymer and avoid the use of complex spring structures. Fiedler et al. [92] deposited a conductive TiN layer on a polyurethane (PU) substrate by using a multiphase DC magnetron sputtering technique (Figure 7c). Thus, the electrode was able to fit the shape of the brain and keep good contact with it. The electrode had a specific structure with 36 electrode pins on a single baseplate. Compared to wet electrodes, the variance of both signals was in the same order of magnitude. In addition, finger-shaped electrodes can be obtained by 3D printing. After the PLA plastic was printed as the mechanical structure, Ag/AgCl ink can be used as a conductive coating. Because of this special processing method, it holds substantial potential for personalized healthcare as shown in Figure 7d [93].

To manage thick hair, there are also brush-like electrodes similar to finger-type electrodes. They have been widely studied for some time because of their good performance. Kitoko’s research [94] provided a method using a reusable silver/silver chloride made with twelve 2 mm contact posts or bristles for EEG testing (Figure 7e). The results did not reveal any significant differences between the two electrodes in all six subjects tested compared to wet electrodes. Grozea et al. [95] proposed a bristle electrode by coating thin polymer bristles with silver particles. This kind of electrode can provide good contact with the scalp and good comfort. However, soft bristles are a double-edged sword. The conductive coatings can very easily to fall off due to the excellent flexibility of the bristles. Gao et al. [96] designed a novel passive dry pin-shaped electrode consisting of a pedestal and bristles (Figure 7f). The pedestal of the electrode was made of CNT-filled polydimethylsiloxane (PDMS-CNT), while the pins were fabricated from carbon fiber and PU with CNT doping. For the bristles, the carbon fiber would not easily break off due to the much higher modulus of elasticity than that of Ag or PDMS. Because of the small contact area of the pointed dry electrode, the subjects will feel uncomfortable. In addition, the coating is likely to fall off due to the flexibility of the probe or bristles. Other special structures that adapt to the shape of the hair and scalp can be made by 3D printing. Lee et al. [97] printed a reverse-curve-arch-shaped dry EEG electrode consisting of 92.5% Ag and 7.5% Cu (Figure 7g). The special shape can ensure the electrode overcomes the obstruction of hair effectively and the shortcomings of the limited contact area and pain induced of finger-shaped electrodes.

Besides the finger shape of pure metal, some methods are similar to conductive silica gel, in which conductive fillers are mixed into the matrix. Compared with the former, this method has a lower cost, and the comfort will be better due to the low modulus of the polymer substrate. There is no need for a complicated spring structure to adapt to the curvature of the scalp, but the flexibility of the material itself can adapt to the shape of the skull. Specifically, Krishnan et al. [98] embedded carbon fiber into silicone foam owing to the biocompatibility and chemical inertness, which are appealing for use in EEG systems. Considering the conductivity, hardness, flexibility, and ease of fabrication, Chen et al. [99] mixed an ethylene propylene diene monomer matrix with different additives such as carbon, stainless fibers, and carbon nanotubes. The skin electrodes’ impedance for 50% carbon content was about 10-fold higher than that of conventional wet electrodes, and the polymer electrodes containing 45% carbon had the best compromise between electrical and mechanical properties. The polymer electrodes showed nearly the same correlation and coherence as the wet electrode signals, but due to the high impedance, a lower SNR was obtained.

In addition, there are some new textile EEG electrodes, also named “textrodes”, that have gradually proved their feasibility in EEG acquisition due to their flexibility, stackability, and washability [61]. The initial use of textile electrodes was for neonatal monitoring systems. For long-term EEG detection in newborns, the electrodes should not stimulate the sensitive skin of infants. However, any type of the former electrodes will inevitably bring about a pressure point. Therefore, textile electrodes came into being. These were originally used with a conductive paste, which suggests that they do not deviate from the category of “wet electrodes”. There are two electrodes proposed. The first one was knitted using a yarn containing 78% polyamide and 22% elastomer. The fabric was plated with 99% pure silver. The second one was made of 15% nylon, 30% silver-plated conductive fibers, 20% Spandex, and 35% polypropylene. The material was knitted and is similar to a terrycloth. However, the author did not consider the performance of the dry electrode after the conductive paste had dried [100]. Similarly, Kumar et al. [101] carried out the same research. Polyethylene terephthalate (PET) fabric was used as a substrate with a copper coating on it. The fabric layer encloses foam to ensure comfort for users. The SEM images show a very uniform deposition of copper on the fabric leading to good conductivity. However, electroless copper plating was carried out through multistep processes, including pre-treatment, sensitization, activation, electroless copper plating, post-treatment to stop copper reduction, rinsing, and drying. Lin et al. [102] proposed a dry foam-based electrode for long-term EEG measurement by selecting urethane material as the matrix covered with 0.2 mm thick taffeta material made using electrically conductive polymer fabric. Finally, all surfaces were coated with Ni/Cu to establish electrical contact. The foam structure allowed high geometric conformity between the electrode and irregular scalp surfaces to maintain low skin–electrode interface impedance. Silver and copper are very popular among coating materials. In addition to pure metal particle coatings (Figure 7h–j), there are also some ideas using graphene coating [103] for ECG and polyaniline PANI [104].

With the development of integration, there are still some dry electrodes only focusing on the hairless area, which are more lightweight. For example, Li coated Ag/AgCl onto a polyacetylimide substrate by screen printing [105]. Jiang et al. [106] used the electrically conductive composite of Ag flakes and PDMS on the silicon substrate. This method of conductive material attached to the thin flexible substrate is usually used in the hair-free area of the forehead, which can achieve the same performance as that of wet electrodes. With the development of the production process, some circuits including amplifiers and capacitors can be integrated into the substrate. At present, researchers can even combine other functions with EEG electrodes. For example, Lee et al. [107] mixed silver nanowire and carbon nanotubes into PDMS (polydimethylsiloxane), forming an elastomeric conductive electrode that also acted as an earphone (Figure 7l). Kappel et al. [108] adopted a thermal iridium oxide film (TIROF) formed on an etched Ti surface as an electrode, and inserted it into holes in a soft earpiece as shown in Figure 7m.

Flexible and composite materials are a current development trend in dry electrodes. These properties can maintain the electrodes’ high geometric conformity with the irregular scalp surface and are more portable. By this method, EEG acquisition and other functions can be integrated, which can also release the potential of BCI application as much as possible.

### 2.4. Semi-Dry Electrodes

Recently, many researches have focused on the combination of dry electrodes and wet electrodes. Wet electrodes need long-term preparation and even hair-removal treatment before EEG recording and can cause discomfort during the measurement. For dry electrodes, the electrolyte between the scalp and electrodes is only provided by sweat and moisture in the air. The lack of electrolytes leads to a high impedance, so there is still a gap in the signal quality between dry electrodes and wet electrodes. A semi-dry electrode overcomes the shortcomings of a dry electrode and a wet electrode. The electrode itself contains electrolytes and gradually releases them during use. The electrolytes permeate the stratum corneum and reduce impedance. Semi-dry electrodes avoid long preparation times and high impedance.

In 2013, Mota et al. [109] first proposed the concept of semi-dry electrodes. The polymer-based electrode can release a small amount of a moisturizing agent (30 μL) from a reservoir inside the electrode. Thermoset PU coated with an Ag/AgCl chemically deposited layer was chosen to prototype the electrode concept. The experiments showed that the signal quality of semi-dry electrodes is comparable to that of wet electrodes. However, there are still some problems with them. Semi-dry electrodes need to apply pressure to promote the release of electrolyte liquid, and it is difficult for each electrode to achieve uniform pressure. Thus, the uncontrollable release of electrolyte liquid will result in an unstable signal. Like bristle electrodes, the Ag/AgCl layer of semi-dry electrodes can easily to fall off due to the constant pressure. Li et al. [110] proposed a ceramic-based semi-dry electrode and solved this problem. The electrode was composed of five porous Al_2_O_3_ ceramic pillars, a built-in reservoir, and a sintered Ag/AgCl electrode. Due to the capillary of the porous ceramics, the saline solution can be released continuously at the rate of 10~20 μL/h, which leads to a more stable property.

To make the electrolytes more evenly distributed in each electrode, Hua et al. [111] designed a flexible, multi-layer, semi-dry electrode consisting of three layers: the electrode body layer, the reservoir layer, and the foam layer. At the bottom of the reservoir, there are five leakage holes which are 0.25 mm in diameter so that the electrolyte solution can flow into the foam. The foam layer stores the electrolyte so it does not rapidly drain. The electrode body layer was made of flexible conductive composite materials with PDMS as the distributed matrix and silver nanoparticles as the conductive filler. The conductive foam was coated with Cu and Ni nanoparticles to obtain good electrical conductivity. The structure was suitable for EEG monitoring in some hairy areas, and the correlation with wet electrode signals could reach about 94.25% and 90.65%, respectively, in static and dynamic EEG monitoring. In a recent study, the combination of a bristle electrode and a semi-dry electrode was carried out by Gao et al. The bristle was fabricated using a hydrophilic material so that water could spread from the roots to the top [112]. Similarly, Xing et al. [113] proposed a micro-seepage electrode with five components. There are flexible and elastic brush pen-like tips made of PU that pass through the hair and contact the scalp. After contact with the scalp and with little pressure, the tip can transport electrolytes that were stored in a cavity made of a polymer sponge. Furthermore, this study investigated the contact impedance of the electrode concerning contact pressure on the occipital area. Under different pressures, the contact impedances ranged from 25 kΩ to 8 kΩ (@ 10 Hz) and from 0.3 N to 10 N.

The abovementioned semi-dry electrodes were made using some special structures, but they all had some common problems including complex molding processes and large volumes. Besides this method, there are some other approaches such as conductive sponges or conductive gels. To reduce the volume of an electrode, Peng et al. [114] proposed a novel electrode consisting of a thin piece of porous Ti as a signal and a reservoir made by PDMS for storage of the electrolytes. Resulting from the excellent mechanical performance and biocompatibility, porous Ti is widely used in medical fields. The electrode was the same size as a coin. Lin et al. [115] coated silver nanowires on a melamine sponge with a PVB solution as an accessory ingredient for the adhesion between the melamine sponge and Ag nanowires and the protection of the exposed Ag nanowires. Besides low contact impedance (<10 kΩ) and a high BCI accuracy of the new electrode (86%) compared to that of wet electrodes (88%), the flexible sponge framework and self-locking Ag nanowires combined to give the new electrode remarkable mechanical stability. The method of preparing a conductive sponge is similar to that of the “textrode” mentioned above, in which the conductive material is coated on a soft substrate, but the sponge has better water storage performance, so the conductive sponge can realize the dual functions of water storage and conductivity. In addition, the soft structure of the sponge can give electrodes better geometric conformity with the scalp. Therefore, it is a very promising way for sponge structures to be coated with conductive materials.

The above semi-dry electrodes all had a reservoir to store the electrolyte solution or water. Another method for semi-dry electrodes is to store as many electrolytes as possible in the electrode body. Toyama et al. [116] proposed a solid-gel electrode that was made of CMC sodium salt, calcium chloride dihydrate, glycerol, and pure water. CaCl_2_ and glycerol are water-absorbing materials that provide a superior wettability of the solid gel. For the electrodes to pass through the hair, Qin et al. [65] proposed a finger-shaped electrode composed of an ionic hydrogel, which was a polyacrylamide (PAAm) hydrogel containing NaCl. The contact impedance was far from that of the wet electrodes (17.4 kΩ to 4.2 kΩ). However, the soft ionic hydrogel-based electrodes had a similar performance to that of the conventional wet gel electrodes in terms of the short-circuit noise, EEG signal quality, and the SSVEP user-centered test.

**Figure 8:**
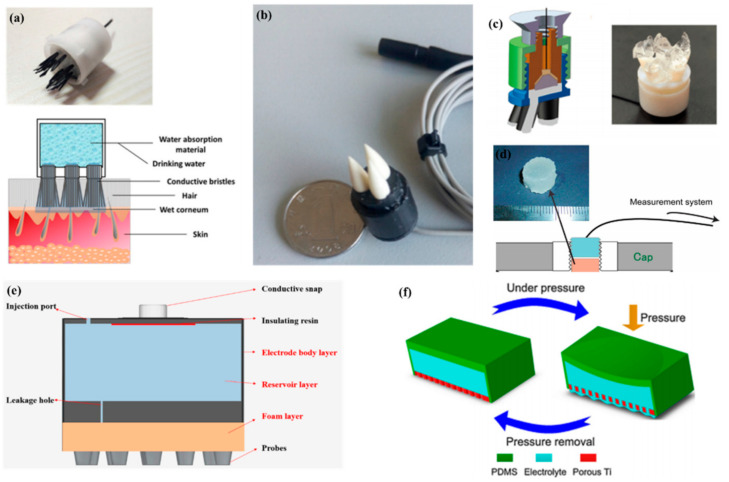
Typical semi-dry electrodes. (**a**,**b**) Semi-dry electrodes with a electrolyte reservoir [112,113]; (**c**,**d**) ionic hydrogel electrodes [65,116]; (**e**,**f**) sponge-based electrodes [111,114].

A common feature of semi-dry electrodes is that they reduce skin contact impedance through the storage and release of electrolytes as shown in Figure 8. Since they have only recently been developed, there is still much room for improvement, such as how to ensure that the electrodes can easily pass through the hair and release electrolytes, but still maintain a good sense of comfort.

## 3. Evaluation Methods for EEG Electrodes

Table 1 shows performance comparisons of some EEG electrodes. Skin contact impedance and correlation are important performance evaluation criteria, and are currently the most common performance that can be directly compared. But many other properties that should be paid attention to. In this chapter, we provide a reference for the current chaotic performance evaluation of EEG electrodes in some aspects.

### 3.1. Evaluation of Chemical Characteristics

As most of the literature about brain electrodes is not based on the perspective of materials, many types of research on BCI do not focus on the electrode itself, but the electrodes deserve attention. The first problem is the purity of electrode materials. At present, most brain electrodes are purchased from commercial companies directly, and few of them are manufactured by BCI researchers, which leads to the failure in explaining the conductive mechanism from the intrinsic view. The morphology and phase composition should be determined from the material characterizations of SEM and XPS, etc., and then the corresponding characterization should be carried out for different materials. The bonding of the polymer electrode is determined by FTIR and NMR. Raman could be used to determine the degree of regularity of carbon structures for brain electrodes based on carbon materials, while for composite materials, TEM characterization of the surface interface connection and EDS characterization of the uniformity are required.

### 3.2. Simulation of Actual Application Scenarios

Nowadays, most EEG electrode performance tests still remain in the laboratory with using the latest prepared electrodes. In the case of the practical application of samples as products, many complex situations should be considered, including consideration as to what will take place to the electrodes after long-term use, whether the original performance can be maintained in the complex environment of the human body, and whether there should be some special care for some special types of electrodes. These electrodes need to go through a series of tests from laboratory samples to practical commercial products.

#### 3.2.1. Antioxidant Performance

In the actual application process, since the electrode is exposed to air for a long time, it is necessary to consider the performance change in the electrode after oxidation. Oxidation directly deteriorates the intrinsic structure and electrical performance of the electrode. As a result, good oxidation resistance can not only extend the service life of the electrode and reduce the cost but also reduce the damage of the electrode to humans. However, oxidation is usually a relatively long process. To speed up this process, the electrodes are usually placed in a higher temperature environment for a while before testing their final performance, such as changes in impedance and chemical properties, to infer the oxidation status. Irrespective of the kind of electrode, they need to undergo this kind of rigorous and extreme testing. The electrode should be put into a furnace with air atmosphere at a temperature that will not directly damage it, such as 60 °C. After a few days of treatment, the change in its impedance and the change in its surface composition will show its oxidation resistance.

#### 3.2.2. Sweat Resistance

Human and animal sweating is a physiological phenomenon just like the water evaporated by the photosynthesis of plants. The main components of sweat are H_2_O, sodium chloride, and a small amount of urea, lactic acid, and fatty acids. The pH value is generally 4.2 to 7.5, which is basically acidic. Some electrode materials may produce some chemical reactions in an acidic medium, so it is necessary to simulate the situation of sweat before use. Similar to the test method in Section 3.2.1, the electrode is placed in the configured artificial sweat, and after a few days of soaking it is observed whether its performance can be maintained as before. Furthermore, silver, which is widely used in the field of brain electrodes, will tend to react with sulfur to produce silver sulfide. Some specific materials are also extremely sensitive to specific ingredients. This is a point worth noting.

#### 3.2.3. Moisture Retention

For semi-dry electrodes and wet electrodes, long-term endurance has always been criticized. As time goes by, the conductive paste of the wet electrode and the saltwater stored in semi-dry electrodes will gradually evaporate, resulting in a great increase in skin contact impedance. Replenishing water at any time is not in line with it remaining lightweight. The most ideal state is to use hydrophilic materials to continuously absorb water vapor and sweat in the air used as electrolytes during use. It is quite difficult to measure the skin contact impedance of the same electrode at a fixed interval, so the measurement for the electrode itself may be a good alternative. Under the conditions of a temperature close to human body temperature (37 °C) and suitable humidity, the weight loss rate or impedance change R − R_0_/R_0_ of the salt water electrode can be measured at different time intervals to determine how long the effectiveness can be maintained in actual use.

#### 3.2.4. Structural Stability

Some electrodes are manufactured by plating a conductive coating on the substrate. However, during use, mechanical deformation and friction are very likely to cause these coatings to fall off. As a result, the break in the conductive path will lead to high impedance. Therefore, there should be a certain number of cycle stress tests. After that, the impedance of the electrodes or element detection is required in order to evaluate the dropped items [115,117]. At present, only few articles mention this.

### 3.3. Electrochemical Performance

#### 3.3.1. Impedance

The first performance is impedance, including the impedance of electrodes and skin contact impedance.

The impedance of electrodes may not be as important in practical applications, and it is meaningless to compare the impedance of different types of electrodes, but the change in impedance is the intuitive embodiment of the change in electrode properties affected by the environment. At present, there is no standard method by which to measure impedance. The more persuasive method is to stick copper foil on both ends of the electrode and then clamp the fixture on the copper foil. Because the principal component of EEG signals is below 100 Hz, the test frequency is usually set at 10 Hz.

Low skin contact impedance is an important guarantee for obtaining better EEG signals. Skin contact impedance is related to many factors. The contact impedance will decrease as the area of the electrode contacting the skin increases. Therefore, in the elaboration of impedance values, area normalization should be carried out. The concentration of the electrolytes used in semi-dry electrodes will have a certain influence on the impedance. Therefore, during the test, the concentration and type of electrolyte should be specified. Additionally, one must ensure that the electrolyte concentration is not too high in order to avoid dehydration of the skin. Due to differences in skin conditions, the impedance of different tested individuals will also vary greatly, even with several-fold greater differences. In addition, there is also a difference in impedance between hair-rich areas and hairless areas. These factors make it very difficult to formulate standards for skin contact impedance. Recently, some studies have used artificial models as simulated skin to formulate impedance standards, such as bilayer agarose gels and gelatin [118,119]. Although these models are incapable of fully simulating the state of the skin, it is still a good start.

#### 3.3.2. Electrode Polarization and Electrochemical Noise

EEG signals have a weak potential of about 1~200 μV. When the metal electrode contacts the electrolyte, the electrode potential will be generated, which is much bigger than the EEG amplitude. According to the Nernst equation,

(1) $$E_{hc}=E_{0}+\frac{RT}{nF}ln\frac{a_{ox}}{a_{red}}$$

*E*_0_ is the standard electrode potential relative to the standard hydrogen. For different electrode materials, *E*_0_ is different. If the electrodes of the same materials contact the same electrolyte, their half-electrode potential *E_hc_* is identical in theory. Therefore, the electrode potential has no interference on the output of the differential amplifier. However, any tiny difference between the two electrodes will cause a large offset voltage, which is much bigger than the EEG. Thus, the composition of the electrodes and electrolytes should be highly consistent, and the electrodes need a stable potential.

Moreover, electrode polarization is another problem that will cause a great error in the electrode potential. When the current flows through the electrode/electrolyte interface, the electrical potential changes from the equilibrium potential to a new electrode potential, which is related to the current density. The non-polarization performance is necessary for electrode materials. In addition, the polarization performance of the electrode can be determined by testing the OCP (open circuit potential) [53,105,110,113]. A smaller potential drift during use is necessary to obtain good EEG performance, so this part of the electrical performance should also be taken seriously.

Besides the electrode polarization, electrochemical noise refers to the fluctuation in electrode potential and current caused by an electrode interface reaction, which will cause a great impact on the EEG signal. In general, this is usually measured by placing two electrodes on the polished metal plate [65,112] or the conductive electrolytic gel [63] to obtain the power spectral density (PSD).

### 3.4. Mechanical Performance

For MEMS electrodes, it is necessary to ensure that the electrode can pierce the cuticle, so the material should have a higher modulus and strength. But for the ordinary-contact electrode, the electrode only needs to ensure that it can pass through the hair and touch the scalp, so there are no strict requirements for the modulus and strength. For some dry electrodes, some innovative structures with springs and spongy dry electrodes are used. They should be tested for thousands of load cycles to prove that they can also guarantee the same performance as the original ones under long-term and repeated use.

### 3.5. Biocompatibility

Although EEG electrodes do not directly contact the brain tissue, unlike invasive electrodes, to ensure minimum harm to the human body, they also need to ensure good biocompatibility. Many types of research are only a summary of the biocompatibility of the material, but in practical applications there is no actual proof of it [53,66,91,108]. Under the ISO standard, certain materials may be biocompatible, but under the condition of EEG acquisition, the electrode will inevitably have some subtle changes, which will affect the human body. EEG electrode materials should be completely harmless to the human body, and all brain electrode researchers should pay attention to health problems. The conductive gel of wet electrodes and the electrolyte of semi-dry electrodes should also be considered in terms of biocompatibility.

### 3.6. Operation Difficulty and Comfort

For the industrial application of BCI, simple daily operation processes and wearing comfort are essential. The pre-operative preparation of wet electrodes, including skin pre-treatment and the application of conductive paste, is very inconvenient in practical application. This is also an important reason why wet electrodes are gradually being abandoned by commercial BCI. The closer contact of electrodes will greatly reduce skin contact impedance, but it will also make the skin feel painful, so researchers should find a balance between the two factors. Additionally, some researchers have conducted questionnaire surveys on these subjects to prove that the comfort performance of their electrodes is acceptable [99,113,120]. I think this is a good start, but there should be a more objective evaluation system, such as the intensity of pressure on the skin or the total weight of the EEG collection system.

Most of the properties mentioned above do not have a clear parameter standard, and the performance of the electrodes cannot be directly compared. However, I think that in the field of brain electrodes, these properties should be taken into account in the preparation of electrodes.

## 4. Challenges for EEG Electrodes and Expectations

For invasive electrodes, if the electrode size is too large, it will compress the nerves, but reducing the electrode area will cause extremely high impedance. The integrated design of materials and structures should be studied to avoid an immune response after implantation. In addition, there is a high risk of infection with invasive/semi-invasive technology, and surgical implants are expensive and complicated. Safety is still a major problem for invasive/semi-invasive technology and MEMS dry electrodes.

As the gold standard, the wet electrode has the advantages of low impedance and non-invasiveness, so it is still in an irreplaceable position in practical applications. The installation of an electrode cap is a time-consuming preparation procedure requiring professional skills and a tedious process; consequently, the test environment is limited. In addition, the gradual evaporation of the conductive gel will affect the stability of the electrode. The original function of the conductive gel is to pass through the hair so that the electrode could contact to the scalp directly, but the conductive adhesive will cause skin irritation, and too much conductive adhesive may form conductive bridges between adjacent electrodes causing a short circuit. Finally, there will be an uncomfortable removable after use. Even so, wet electrodes are still the most frequently used methods in the laboratory.

Dry electrodes have several forms with corresponding advantages, but none of them need any conductive medium, so the same characteristics save the complicated preparation work before the acquisition of the EEG signal and the cleaning steps after the acquisition. An urgent problem of dry electrodes is the reduction of its skin contact impedance. Therefore, to achieve this goal, increasing the contact pressure has proven to be an effective method that not only allows the electrodes to pass through the hair but also thins the stratum corneum after squeezing the skin. However, under the premise of ensuring comfort, it is a difficult to ensure the appropriate modulus to pass through the hair.

Semi-dry electrodes have become a hot spot in current EEG acquisition research, and they have several forms including specific structures, gels, and sponges/woven. Compared with wet electrodes, they do not require cumbersome steps. Compared with dry electrodes, due to the existing electrolytes, the impedance of the stratum corneum is greatly reduced. With the emergence of new materials, advanced structures, and improving processing technology, semi-dry electrodes have the potential to meet the long-term daily monitoring and measurement requirements.

The materials used for electrodes should also be paid attention to. Good conductivity and biocompatibility are the most basic requirements. The stability and processability of materials should not be ignored. In addition to the type of electrode itself and the materials used, the structure is also very important. Maintaining a high geometric conformity is essential for collecting good EEG signals. Therefore, it has become a trend to use flexible materials as substrates, but there are other methods, such as multipin electrodes.

In the preparation process of all the above electrodes, we should also pay attention to the quality of some aspects of performance. We noticed that in the electrode performance tests of researchers, most of the emphasis was only on the elaboration of the EEG signal, while the issues of biocompatibility, electrochemical noise, and so on mentioned in Part 3 should also be taken seriously. In addition, to avoid occasionality, there should be as many subjects as possible participating in the live tests, and the sensor location should conform to the international standard.

For patients, medical staff, and digital enthusiasts, the more secure and portable BCI devices are more acceptable. With the advancement of integration technology, the reception and processing of EEG signals can be integrated into a smartphone, but the acquisition of signals still restricts the wide application of BCI. It is believed that with the improvement of signal acquisition technology, the application of the brain–computer interface will reach a new stage. The way of life of mankind will also be greatly innovated.

## Figures and Tables

**Table 1:** Materials, structures, and properties of some EEG electrodes.

Electrode Type	Materials (Structure)	Contact Impedance	Correlation	Ref.
MEMS electrodes	Ti/Pt @ Si substrate	—	83~86%	[70]
	Ti/Ag @ Si substrate	12.5 kΩ~20 kΩ (@ 10 hz)	91.63% (@ forehead)	[72]
	Au/SU-8 @ Ti substrate	40 kΩ (@ 10 hz on the inner forearm)	—	[74]
	Cu	1.9 kΩ (@ 50 kHz on the inner forearm)	—	[75]
	IrO	Lower than Ag/AgCl wet electrode	—	[78]
	Ag flakes in silicone	—	97.85%	[79]
	Ag @ flexible polyimide organic layer	3 kΩ (@ Fp_1_) and 2.7 kΩ (@ Cz)	—	[80]
Non-contacted electrodes	A layer of 30 μm polyimide, 30 nm titanium, 10 μm Cu, 30 μm Ni and 100 nm Au	—	91% (eye closed) and 83% (eyes open)	[86]
	Cu	—	92.05%	[88]
	Cu	—	—	[87]
Common-contact electrode	BeCu plungers coated with Au	9 kΩ (@ forehead) 16 kΩ (@ hariy sites)	95.26% (@ forehead) and 91.47% (@ hairy sites)	[66]
	Spring probes coated with a platinum nanoporous layer	11.5 ± 4.9 kΩ	81.79~96.77%	[91]
	PU multpin coated with TiN	65~76 kΩ (@ Fp_2_)	—	[92]
	Fingered PLA plastic coated with Ag	3 kΩ (@ 10 hz)	86.2~99.5%	[93]
	Bristles made of Ag/AgCl	5~10 kΩ	—	[94]
	Bristles coated with Ag	80 kΩ	—	[95]
	Pin-shaped PDMS embedded with carbon fiber and coated with Au	13 kΩ~417 kΩ (Average 133 kΩ)	>90% at most of the frequencies	[96]
	Reverse-curve arch made of 92.5% Ag and 7.5%Cu	70 kΩ (@ forehead) and 125 kΩ (@ hairy sites)	—	[97]
	Fingered EPDM embedded with carbon fiber stainless steel fiber and CNT (finger-shaped)	—	90%	[99]
	Ti/TiN	About 250 kΩ	—	[53]
	PU foam coated with Ni/Cu	7 kΩ~15 kΩ (0.5 Hz~1000 Hz on the forehead)	95.56% (@ forehead)	[17]
	TYPE I: A yarn containing 78% polyamide and 22% elastomer and plated with 99% pure silver TYPE II: 15% nylon, 30% silver plated conductive fibers, 20% Spandex and 35% polypropylene.	—	82~88%	[100]
	PU foam with electrically conductive taffeta fabric and Ni/Cu coating	9 kΩ (@ forehead) and 16 kΩ (@ hairy sites)	96.14% (@ forehead) and 90.12% (@ hairy sites)	[102]
	PU foam coated with PANI	—	—	[104]
	Ag/AgCl screen printed on a sweat-absorbable sponge layer	2325 ± 1025 Ω (wet skin) and 36,366 ± 17,286 Ω (dry skin)	90.8 ± 6.2% (dry skin) 96.2 ± 3.2% (wet skin)	[105]
Semi-dry electrodes	PAAm hydrogel containing NaCl	17.4 kΩ	93.65% (@ F10) and 95.64% (@ Pz).	[65]
	Thermoset PU foam coated with an Ag/AgCl chemically deposited layer	—	61~94%	[109]
	Plungers made of Al_2_O_3_ porous ceramic	22.2 ± 8.5 kΩ	93.8 ± 3.7%	[110]
	Silver nanoparticles distributed in PDMS matrix	18.18 ± 7.51 kΩ (@ Fpz) and 23.89 ± 7.44 kΩ (@ Oz)	90.65~94.25%	[111]
	Nylon coated with carbon	15 kΩ	90.89% at FCz, 92.61% at Cz and 92.62% at Pz	[112]
	PU foam	25 kΩ to 8 kΩ (@ 10 Hz) from 0.3 N to 10 N	—	[113]
	Porous Ti	2.4 kΩ on forehead 10 hz	95.55% (semi-dry) and 90.18% (dry)	[114]
	Melamine foam coated with Ag nanowires	<10 kΩ	—	[115]
	A solid-gel electrode containing CMC sodium salt, calcium chloride dihydrate, glycerol, and pure water.	From 3 to 25 kΩ (typically 10 kΩ)	—	[116]
